# Effects of N Fertilization During Cultivation and *Lactobacillus plantarum* Inoculation at Ensiling on Chemical Composition and Bacterial Community of Mulberry Silage

**DOI:** 10.3389/fmicb.2021.735767

**Published:** 2021-10-07

**Authors:** Ping Li, Minghong You, Zhouhe Du, Yongxiang Lu, Chunyan Zuo, Man Zhao, Honglin Wang, Xu Yan, Chao Chen

**Affiliations:** ^1^College of Animal Science, Guizhou University, Guiyang, China; ^2^Grass and Forage Research Institute, Sichuan Academy of Grassland Sciences, Chengdu, China; ^3^Sericultural Research Institute, Sichuan Academy of Agricultural Sciences, Nanchong, China

**Keywords:** mulberry, N fertilization, silage, high throughput sequencing, bacterial community

## Abstract

As unconventional forage source, mulberry (*Morus alba* L.) has been cultivated to alleviate animal feed shortages. This study aimed to investigate the effects of N fertilization during cultivation and *Lactobacillus plantarum* inoculation at ensiling on the chemical composition and bacterial community of mulberry silage. Mulberry was separately cultivated under two N fertilization rates (N1, 390 kg/ha/year; N2, 485 kg/ha/year) in 2016–2019, harvested on 30 April (the first-cut) and 15 June (the second-cut) in 2019, and then chopped for producing small bag silage. The silage was treated without (control) or with *L. plantarum* (LP, a recommended application rate of 10^5^ cfu/g on fresh matter basis). After storage of 60 days in dark room at ambient temperature, silage was sampled for analysis of chemical and microbial compositions. Higher (*P* < 0.05) final pH value and acetic acid content and lower (*P* < 0.05) lactic acid content were found in silage of mulberry under N2 fertilization, resulting in more dry matter loss than that under N1 fertilization. Compared with control, inoculation of LP at ensiling increased (*P* < 0.05) lactic acid content and decreased (*P* < 0.05) final pH value, acetic acid and propionic acid contents of silage, by advancing the dominance of *Lactobacillus* and reducing the abundance of *Enterococcus* and *Enterobacter*. In particular, inoculation of LP at ensiling decreased (*P* < 0.05) dry matter loss and butyric acid content of first-cut silage. In conclusion, inoculation of LP at ensiling could reduce the undesirable effects from high N fertilization rate during cultivation on silage quality of mulberry harvested at different growing seasons.

## Introduction

Mulberry (*Morus alba* L.) is traditionally cultivated for the silkworm production industry in the world (Sánchez, 2000; Zhou et al., 2014). In past decades, mulberry has been used as an unconventional feed in ruminant and non-ruminant production systems (González-García and Martín Martín, 2017), due to its high digestibility, crude protein content and energy and low cell wall content (Kabi and Bareeba, 2008). In addition, mulberry contains no anti-nutritional factors or toxic compounds (such as tannins or saponins that occur in many legume forages) and high levels of bioactive compounds (such as polysaccharides, polyphenol, and essential amino acids) in leaves and young stems, which are beneficial to animal and subsequently human health (Wang et al., 2012; Zhao et al., 2015). However, how to best preserve the nutrients of mulberry at growing seasons is a concerned issue.

Agricultural practices influence field performance, nutritional components and inherent/epiphytic microbial population of forage at harvest, subsequently determining the ensiling process and silage quality. In particular, high N fertilization rate in high-yield cultivation system increases buffer capacity and/or decreases water soluble carbohydrates of crops, which reconstructs bacterial community and increases final pH value and ammonia-N ratio of total N in grass silage (Tremblay et al., 2005; King et al., 2013; Jahanzad et al., 2014; Lv et al., 2017). A study from Namihira et al. (2010) also indicated that tropical-grass silage fermentation pattern and quality resulted from the differences in microbial communities between the different N fertilization levels. Thus, effects of N fertilization rates during cultivation on silage fermentation and bacterial community of mulberry is worth exploring.

Next generation sequencing could provide a detailed picture of microbial community of silage. Studies from He et al. (2019) and Wang et al. (2019) have described the bacterial community of mulberry leaf silage. However, short sequences with a relatively low taxonomical resolution have limited the classification of microorganisms in the community to the genus level (Amir et al., 2013). Recently, the third generation PacBio single-molecule, real-time (SMRT) sequencing was used to depict the bacterial community composition of ryegrass and oat silages, due to its increased sensitivities and accuracy of classification to species level (Yan et al., 2019; Chen et al., 2020; Du et al., 2021). It is helpful to apply the PacBio SMRT sequencing to better explore the bacterial community composition and species diversity of mulberry silage.

In addition, additives were used in production of mulberry silage, mainly including lactic acid bacteria (LAB) inoculants (*Lactobacillus plantarum* or *Lactobacillus casei*), sucrose, cellulase, propionic acid, and gallic acid (He et al., 2019, 2020; Wang et al., 2019; Zhang et al., 2019). However, little information is on additive-treated silage of mulberry under different N fertilization rates. Therefore, an experiment was conducted to investigate the interactive effects of N fertilization during cultivation and LAB inoculation at ensiling on the chemical and microbial compositions of mulberry silage. Inoculation of *L. plantarum* at ensiling is widely used in preservation of silage nutrients, because it could compensate undesirable effects from agricultural practice in silage production. We supposed that (1) high N fertilization rate during cultivation exerted a negative effect on preservation of silage nutrients; (2) *L. plantarum* inoculation at ensiling enhanced the fermentation of mulberry silage by shifting bacterial community.

## Materials and Methods

### Experiment Site and Forage Cultivation

Six filed plots (3 m × 5 m) were designed to cultivate mulberry (Chuansisang No. 1) at the experimental base (E 106°12′, N 31°11′; altitude, 280 m) of Sericultural Research Institute, Sichuan Academy of Agricultural Sciences, Nanchong, China, in 2016. The soil is featured by pH of 7.5, organic matter of 42.8 g/kg, available N of 178.8 g/kg, available K of 163.7 g/kg and available P of 11.9 g/kg. Mulberry plant density was 105,000 plants/ha. In March of 2017∼2019, three of the plots were fertilized with N1 (N:P:K = 390:500:132, kg/ha/year), and the others were fertilizer with N2 (N:P:K = 485:500:132, kg/ha/year). Urea, calcium magnesium phosphate, and potassium sulfate were used as N, P, and K fertilizers. N and K fertilizers were separately divided into four equal portions; the first portion was fertilized after sprouting of mulberry, and the other portions were fertilized equally after the first-, second-, and third-harvests of mulberry; there was no fertilization after the fourth harvest of mulberry. P fertilizer was divided into two equal portions, and separately applied after the second- and fourth-harvests of mulberry. Irrigation was timely conducted when no rain occurred for more than 2 weeks. Each plot was weeded manually in time after each harvest of mulberry forage. The fresh yield of mulberry in 2017–2019 was 74.45–84.93 t/hm^2^, with stem-to-leaf ratios of 0.55–0.72. The first 2 year-data on field performance and chemical components of forage mulberry was partly reported by Wang et al. (2020).

### Silage Preparation

Mulberry from each plot was harvested on 30 April (the first-cut) and 15 June (the second-cut), 29 July (the third-cut), and 29 September (the fourth-cut) in 2017–2019, respectively. Preliminary data (not shown in article) in 2017–2018 illustrated that good fermentation occurred to the second- and third-cut mulberry silages. In 2019, thus, the first-cut and second-cut mulberry in each plot was chopped to a length of 1.0–2.0 cm, and randomly divided into 2 equal portions for additive treatments. The ensiling materials were treated with no additive as control (CK) or with *L. plantarum* [LP, a recommended application rate of 10^5^ cfu/g on fresh matter (FM) basis, isolated from natural fermented-silage in our laboratory]. Each silo bag contained approximately 1 kg (FM) of forage mulberry, and a total of 24 silo bags (2 cut × 2 N fertilization × 2 treatments × 3 replicates) were sealed under the anaerobic condition, stored at room temperature for 60 days, and then sampled for chemical and microbial analysis.

### Chemical Composition Analysis

Each sample of 20 g was mixed with 180 mL sterile water, mixed homogeneously for 1 min in a laboratory juicer, and then filtered through four layers of cheesecloth. The filtrate was subjected to centrifugation (4500 × *g*, 15 min, 4°C). The supernatant was used to measure pH, ammonia-N, and organic acid. The pH was determined by pH meter. Ammonia nitrogen (NH_3_-N) was determined by the method of Broderick and Kang (1980). Lactic, acetic, propionic, and butyric acids were analyzed using high-performance liquid chromatography (Li et al., 2019).

Each sample of about 300 g was dried at 65°C to a constant weight to determine dry matter (DM) content, and then ground through 0.20 mm sieve for analysis of crude protein (CP), neutral detergent fiber (NDF), acid detergent fiber (ADF), and water carbohydrate (WSC). CP was determined by the method of AOAC (1990). Both NDF and ADF were determined using an ANKOM 2000 fiber analyzer (ANKOM Technology, Fairport, NY, United States) by the method of Van Soest et al. (1991). WSC was determined by the method of McDonald et al. (1991).

### Microbial Composition Analysis

Microbial population in each sample was determined by the method of Cai et al. (1999). In brief, each sample of 10 g was mixed with 90 mL sterile saline, shaken for 30 min and then filtered through sterile gauze. Serial dilutions were produced. Both MRS agar and nutrient agar medium from Land Bridge Technology Co., Ltd., Beijing, China were incubated at 37°C for 48–72 h, to separately count LAB and aerobic bacteria. Enterobacteria was counted on Violet Red Bile (HBO116-5, Qingdao Hope Bio-Technology Co., Ltd., Qingdao, China) incubated at 30°C for 36 h. Yeasts were numbered on malt extract agar with 1.5 mg/L Tetracycline (CM173, Land Bridge Technology Co., Ltd., Beijing, China), incubated at 28°C for 48 h. Yeasts were distinguished from molds based on colony appearance and cell morphology. Counts of LAB, aerobic bacteria, enterobacteria and yeasts of each silage sample were estimated as log_10_ cfu/g of FM.

The silage bacterial community analysis based on third generation sequencing technology was described by Chen et al. (2020). The total DNA from each sample was extracted by Mag-MK Bacterial Genomic DNA Extraction Kit (Sangon Biotech, Shanghai, China). DNA after purification was diluted to 1 ng/mL using sterile water. The full-length 16S ribosomal RNA (rRNA) gene was amplified using specific primer (27F and 1514R) with barcode (Yan et al., 2019). The PCR reaction was carried out with TransStart^®^ FastPfu DNA Polymerase (TransGen Biotech). PCR products were mixed at the equal density ratio and purified with QIAquick^®^ Gel Extraction Kit (QIAGEN). Libraries were generated using SMRTbellTM Template Prep Kit (PacBio) according to the manufacturer’s recommendations, assessed on the Qubit^®^ 2.0 Fluorometer (Thermo Fisher Scientific) and FEMTO Pulse system, and then sequenced on the PacBio Sequel platform. The raw sequences were processed as the procedure stated by Yan et al. (2019). Sequences analysis was performed by Uparse software (Uparse v7.0.1001)^1^. Sequences with ≥97% similarity were assigned to the same operational taxonomic unit (OTU). SSUrRNA Database of Silva Database^2^ was used to annotate taxonomic Information of each representative sequence. After normalization of OTUs abundance information, the alpha diversity indices in each sample were calculated with QIIME (Version 1.9.1) and displayed with R software (Version 2.15.3).

### Statistical Analysis

Factorial analysis of variance was applied to the results with N fertilization (N) during cultivation and additive (A) at ensiling and their interaction (N × A) with harvest-cut as a random effect, in the General Line Model of SPSS (SPSS 25.0 program SPSS Inc., Chicago, IL, United States). Differences were considered significant only when the probability level was lower than 0.05 (*P* < 0.05).

## Results and Discussion

### Chemical Composition and Microbial Population of Fresh Mulberry

As shown in Table 1, high N fertilization rate increased slightly the WSC, CP, NDF, and ADF contents of mulberry. This indicated that high N fertilization facilitated the accumulations of nutritional components of mulberry. Moreover, the chemical composition of mulberry changed between two harvest cuts. Similar situation was found and explained by González-García and Martín Martín (2017) who reported that high temperature and more light in summer advances the maturity of mulberry with more accumulation of photosynthetic products than in spring. Theoretically, the WSC concentration (>3% DM) of mulberry was sufficient for initiating LAB propagation and subsequent lactic acid fermentation during ensiling. In the present study, however, relatively high buffer capacity (257∼352 mEq/kg DM) and distributions of aerobic bacteria (6.17∼7.26 log_10_ cfu/g FM), enterobacteria (4.28∼5.69 log_10_ cfu/g FM), and yeasts (3.63∼4.85 log_10_ cfu/g FM) on fresh mulberry may limit the rate and extent of lactic acid fermentation during ensiling. Thus, fast-increasing dominance of LAB may be effective for producing high-quality mulberry silage.

**TABLE 1:** Chemical composition and microbial population of fresh forage mulberry.

Forage source	*N* level†	DM	WSC	NDF	ADF	CP	Buffer capacity	LAB	Aerobic bacteria	Enterobacteria	Yeasts
						
		g/kg FW	g/kg DM				mEq/kg DM		log 10 cfu/g FM		
First cut	N1	231.0 ± 1.92	68.7 ± 4.8	389 ± 31	193 ± 12	158.2 ± 1.7	341.06 ± 6.85	3.66	7.26	5.38	4.85
	N2	244.0 ± 2.48	78.0 ± 3.1	400 ± 43	214 ± 14	165.5 ± 2.5	352.31 ± 4.14	3.83	7.55	5.69	4.26
Second cut	N1	265.5 ± 2.13	55.2 ± 2.9	438 ± 29	231 ± 13	181.5 ± 2.1	275.22 ± 5.37	4.89	7.42	4.28	3.98
	N2	286.4 ± 1.56	60.9 ± 5.2	445 ± 31	249 ± 15	193.9 ± 2.6	286.37 ± 6.11	5.15	6.17	4.44	3.63
SEM		1.6	2.8	34	11	1.9	5.2	0.08	0.14	0.13	0.20
*P*-value		<0.001	0.021	0.038	<0.001	<0.001	<0.001	<0.001	0.452	0.016	<0.001

*†Application rate of N fertilization during cultivation was N1 (390 kg/ha/year) and N2 (485 kg/ha/year). ADF, acid detergent fiber expressed inclusive of residual ash; AN, ammonia-N; CP, crude protein; DM, dry matter, FW, fresh weight; NDF, neutral detergent fiber assayed with a heat stable amylase and expressed inclusive of residual ash; SEM, standard of error mean; TN, total nitrogen; WSC, water soluble carbohydrates.*

### Chemical Composition of Mulberry Silage

The chemical composition of mulberry silage is shown in Table 2. High N fertilization rate during cultivation increased CP content of second-cut mulberry silage by 9.16∼10.89% in relative to low N fertilization. Similar situation was observed by Macome et al. (2017) who reported that the high N fertilization rate during cultivation increased CP content of ryegrass silage by 32∼63%. However, high N fertilization during cultivation increased DM loss by 7.89∼46.08% for first-cut silage and 17.08∼53.20% for second-cut silage. We speculated that heavy presence of microorganisms such aerobic bacteria, enterobacteria, and yeasts (Table 1) on the fresh mulberry under high N fertilization respired dramatically at early stage of ensiling for a carbon release in form of CO_2_, resulting in an increase in DM loss. Some LAB strains were applied in producing mulberry leaf silage, e.g., *L. plantarum* (Zhang et al., 2019). Under high N fertilization rate, however, inoculation of LP at ensiling increased residual WSC content of silage by 8.23–11.47%. Furthermore, inoculation of LP at ensiling reduced DM loss of silage. These confirmed that inoculation of LP at ensiling was helpful to preserve nutrients of mulberry silage.

**TABLE 2:** Chemical composition of paper mulberry silage.

N level† (N)	Additive‡ (A)	DM	DM loss	WSC	CP	NDF	ADF	LA	AA	PA	BA	Final	AN
						
		g/kg FW	g/kg DM	g/kg DM								pH	g/kg TN
**First cut**													
N1	CK	227c	43.1ab	5.7e	147.7b	398c	205c	23.7ef	11.0b	5.9a	1.3c	4.80b	160.8ab
	LP	231c	21.7c	8.5d	150.4b	401c	204c	32.0c	6.1d	3.9b	0.9c	4.59c	142.1b
N2	CK	234c	46.5a	6.0e	141.5b	407bc	228bc	21.7f	14.9a	5.9a	5.0a	5.04a	181.5a
	LP	246c	31.7bc	8.6d	150.4b	41.1bc	229bc	25.4de	7.9cd	2.2d	2.5b	4.65c	174.3a
**Second cut**													
N1	CK	271b	28.1bc	12.0c	153.3b	458a	243ab	30.9c	13.3a	3.4bc	ND	4.82b	112.7c
	LP	272b	20.3c	21.1b	156.1b	465a	243ab	44.7a	7.9cd	2.7cd	ND	4.36d	98.5c
N2	CK	291a	32.9b	11.9c	170.7a	445ab	256a	27.2d	15.1a	3.1bc	ND	4.90ab	113.2c
	LP	294a	31.1bc	23.6a	170.4a	454a	251ab	41.4b	9.5bc	2.7cd	ND	4.41d	114.5c
SEM		6.0	1.5	2.2	0.63	7.0	6.0	4.8	3.1	0.6	0.3	0.09	7.4
**Significant (*P* < 0.05)**													
*N*		<0.001	<0.001	0.086	0.024	0.926	0.006	0.018	<0.001	0.023	<0.001	0.007	0.002
A		0.611	0.052	<0.001	0.123	0.548	0.834	<0.001	<0.001	<0.001	<0.001	<0.001	0.056
N × A		0.121	0.145	0.099	0.461	0.929	0.836	0.051	0.259	0.099	0.001	0.168	0.168

*†Application rate of N fertilization during cultivation was N1 (390 kg/ha/year) and N2 (485 kg/ha/year).*

*‡Silages treated without (CK) or with *Lactobacillus plantarum* (LP).*

*AA, acetic acid; ADF, acid detergent fiber expressed inclusive of residual ash; AN, ammonia-N; BA, butyric acid; CP, crude protein; DM, dry matter; FW, fresh weight; LA, lactic acid; ND, no detected; NDF, neutral detergent fiber assayed with a heat stable amylase and expressed inclusive of residual ash; SEM, standard of error mean; TN, total nitrogen; WSC, water soluble carbohydrates; PA, propionic acid.*

*Values with different letters in the same column are significantly different (*P* < 0.05).*

Under high N fertilization, silage also exhibited higher levels of final pH, ammonia-N and acetic acid and lower level of lactic acid than that under low N fertilization (Table 2). Similar results were from Namihira et al. (2010) and Lv et al. (2017) who reported that final pH value, lactic acid concentration and ammonia-N ratio of total N were increased in silages of tropical-grass at high N fertilization rates. However, there was little effect of N fertilization on the fermentation degree of perennial ryegrass (*Lolium perenne* cv. Gandalf), Italian ryegrass (*Lolium multiflorum* cv. Prospect), tall fescue (*Festuca arundinacea* cv. Fuego), cocksfoot (orchardgrass, *Dactylis glomerata* cv. Pizza), timothy (*Phleum pratense* cv. Erecta), and corn silages (Lawrence et al., 2008; King et al., 2013). The inconsistence in silage fermentation parameters under different N fertilizations is difficult to explain.

The first-cut silage showed a high level of butyric acid, accompanied by acetic acid formation. Butyric acid is an indicator of undesirable silage fermentation, as it may result in nutrient loss and low feed intake by ruminants (McDonald et al., 1991). The most common (natural) way of inhibiting butyric acid fermentation is the promotion of lactic acid-dominant fermentation to reduce the pH or increasing the DM of material to be ensiled (Namihira et al., 2010). *L. plantarum*, as an important LAB for silage fermentation, is mainly responsible for the simultaneous accumulation of lactate and generally inhibiting the butyrate production of silage. Inoculation of LP at ensiling decreased butyric acid of mulberry silage by 30.77–50.00%. Similar result was observed by Zhang et al. (2019).

### Microbial Composition of Mulberry Silage

**FIGURE 1:**
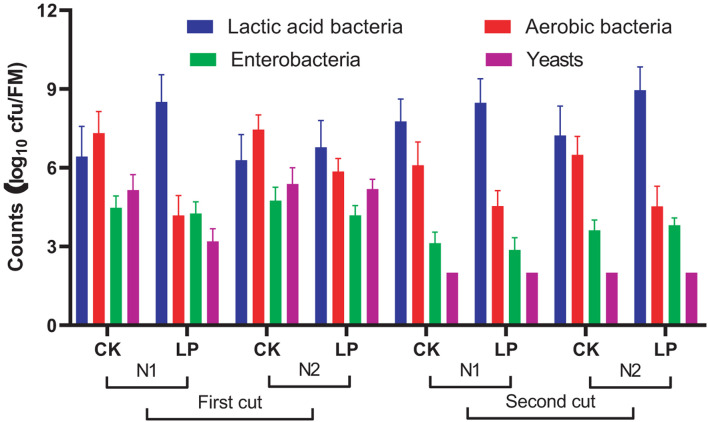
Microbial population of silage treated without (CK) or with *Lactobacillus plantarum* (LP). Application of N fertilizations during cultivation was N1 (390 kg/ha/year) and N2 (485 kg/ha/year). Bar in each column represented standard error of mean.

The fermentation of mulberry silage is determined by microbes during ensiling. According to studies from He et al. (2019) and Wang et al. (2019), LAB and yeasts were predominant populations, and other undesirable microorganisms decreased below the detection limit (<2.0 log cfu/g FM) at the final stage of fermentation in high-moist (>75%) mulberry leaf silage. In the present study, however, mulberry silage exhibited relatively high counts of enterobacteria, aerobic bacteria, and yeasts (Figure 1), which suggested that the fermentation of mulberry silage is incomplete. However, inoculation of LP at ensiling reduced aerobic bacteria population.

Microbial community could reflect the silage quality. In this study, the species diversity and bacterial community composition of LP-inoculated silage of mulberry under different N fertilization rates were reported by the PacBio SMRT sequencing technology for the first time. As shown in Table 3, the coverage of >0.97 indicated that most of bacteria were detected in silage. We observed that the alpha-diversity varied among silage samples. A high number (97∼421) of observed species was found in second-cut silages. A study from Carvalho-Estrada et al. (2020) demonstrated that silage pH is determinant factor affecting bacterial community composition. The pH value of 4.36∼5.04 in mulberry silage mass was relatively high, which did not exert an inhibitory effect on the growth of most bacteria during ensiling. High N fertilization rate increased bacterial diversity index of Shannon and richness index of ACE, which confirmed that relatively high buffer capacity also promote the growth of most bacteria in mulberry silage. Inoculation of LP at ensiling reduced bacterial alpha-diversity indices of mulberry silage. This was in accordance with the results from He et al. (2019); Wang et al. (2019), and Zhang et al. (2019) who reported that the quick pH decline from acid production at the early stage of ensiling accelerated the disappearance of undesirable microorganisms in LAB-inoculated mulberry silage, resulting in a lower bacterial alpha-diversity as compared with control silage.

**TABLE 3:** Bacterial alpha-diversity indices of forage mulberry silage.

N level† (N)	Additive‡ (A)	Clean reads	Observed species	Shannon	Chao 1	Coverage
**First cut**						
N1	CK	5286	44	1.67	45.88	0.998
	LP	7063	19	0.99	22.00	0.998
N2	CK	6619	35	2.85	44.00	0.997
	LP	6539	31	2.70	40.00	0.997
**Second cut**						
N1	CK	5753	125	3.54	166.13	0.984
	LP	6091	97	3.09	140.05	0.987
N2	CK	6442	421	3.96	1267.05	0.985
	LP	5926	244	3.33	594.37	0.974
SEM		23	6	0.04	17.79	0.001

*†Application of N fertilizations during cultivation was N1 (390 kg/ha/year) and N2 (485 kg/ha/year).*

*‡Silages treated without (CK) or with *Lactobacillus plantarum* (LP).*

*SEM, standard of error mean.*

**FIGURE 2:**
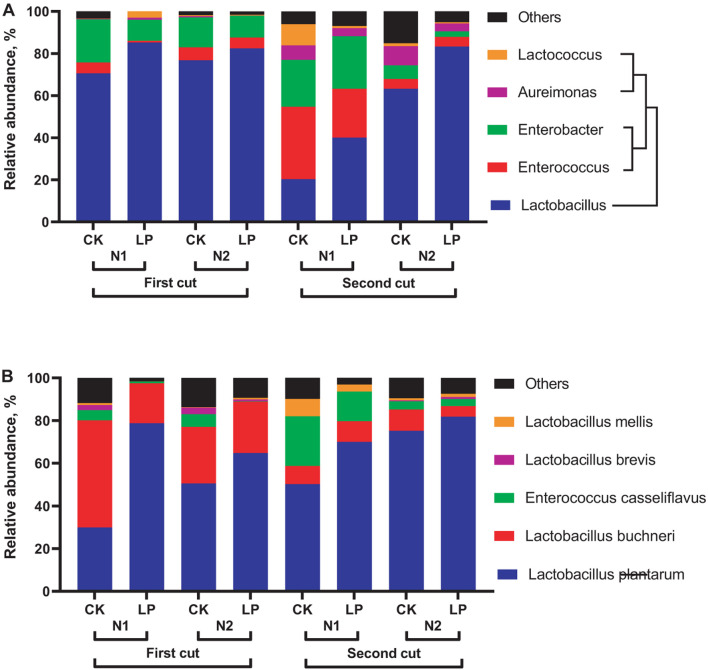
Relative abundance of top five genera **(A)** and species **(B)** in silage treated without (CK) or with *Lactobacillus plantarum* (LP). Application of N fertilizations during cultivation was N1 (390 kg/ha/year) and N2 (485 kg/ha/year).

In the present study, *Lactobacillus* (20.32∼85.27%), *Enterococcus* (0.72∼34.41%), and *Enterobacter* (2.62∼24.93%) were the first three genera in mulberry silage (Figure 2). Wang et al. (2019) reported that the major genera in mulberry silage were *Lactobacillus*, followed by *Exiguobacterium* and *Acinetobacter*, with a total relative abundance of >30%. He et al. (2020) found that *Clostridium* (21.1%), *Kosakonia* (38.3%), and *Lactobacillus* (8.9%) were heavily distributed in mulberry silage. We speculated that differences in the first three dominances of bacterial genera in mulberry silage were determined by ensiling materials, storage period or sampling time in different climate regions (Carvalho-Estrada et al., 2020). As reported by Zhang et al. (2019), inoculation of LP at ensiling of mulberry increased the dominance of *Lactobacillus* by 7.39∼96.99%. However, *Enterobacter* was also heavily distributed in silage, especially for silage of second-cut mulberry under low N fertilization rate, with relative abundance of 23.22∼34.41%. Du et al. (2021) reported that *Enterobacter* and *Lactobacillus* determined fermentation quality of natural fermented-paper mulberry silage. Additives such as gallic acid, sucrose and exogenous LAB could shift the main microbes from *Enterobacter* to *Lactobacillus* (Wang et al., 2019; He et al., 2020). And, a study from Cai et al. (1999) showed that inoculation of homo-fermentative LAB at ensiling promote the disappearance of *Enterobacter* species at the early stage and reduction of *Lactobacillus buchneri* dominance at the late stage of ensiling, resulting in lower acetic acid content of silage relative to control. *Enterococcus* species just occurred at initial stage, and competed out by more acid-resistant LAB at late stage of fermentation in well-preserved silage. Therefore, the high proportion (22.27∼24.39%) of *Enterococcus* species indicates incomplete-fermentation in silage of second-cut mulberry under low N fertilization rate.

**FIGURE 3:**
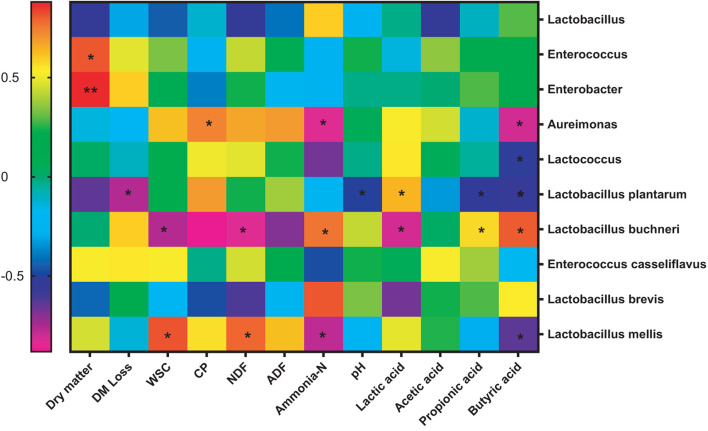
Spearman correlation between chemical compositions and relative abundances of top five genera and species in silage. *P < 0.05, **P < 0.01.

A good correlation between silage fermentation parameters and relative abundance of main microbial species was found in previous study (Du et al., 2021). In the present study, there existed significant (*P* < 0.05) correlations between relative abundance of *Lactobacillus plantarum* and chemical compositions of silage (Figure 3). Positive correlation (*P* < 0.05) in silage was found between DM content and relative abundances of *Enterococcus* and *Enterobacter*. This indicated low moisture in silage mass facilitated the growth of *Enterococcus* and *Enterobacter*, and an incomplete fermentation occurred in the first-cut silage. Under low N fertilization rate, *Lactobacillus* species accounted for a small proportion (<50%) in control silage (Figure 2). It was generally recognized that the increased dominance of *L. plantarum* facilitated the production of lactic acid and the reduction of acetic acid, resulting in a low pH and ammonia-N and/or butyric acid content. Thus, inoculation of LP at ensiling resulted in the increased dominance of *L. plantarum* in silage by 8.78∼163.41%. Similar situation was also observed by Fijałkowska et al. (2020) who reported a high proportion of *Lactobacillus* species in total bacteria was in LAB-inoculated grass silage. In this study, *L. buchneri* as a hetero-fermentative LAB promoted WSC consumption and acetic acid production to increase pH and ammonia-N levels of silage. An opposite trend occurred to *Lactobacillus mellis* in mulberry silage.

## Conclusion

High N fertilization rate during high-density cultivation stimulated accumulation of acetic acid and reduction of lactic acid in mulberry silage, with high pH and ammonia-N levels. SMRT result showed that *Lactobacillus*, *Enterobacter*, and *Enterococcus* were the main bacterial genera in mulberry silage. Inoculation of LP at ensiling enhanced silage fermentation through increasing lactic acid content and decreasing final pH value and acetic acid content. In addition, inoculation of LP at ensiling reduced species diversity and reconstituted bacterial community composition of mulberry silage. Generally, our research confirmed that LP as a microbial additive exhibited high potential for producing and stabilizing high-quality silage of mulberry under different N fertilizations.

## Data Availability Statement

The raw data supporting the conclusions of this article will be made available by the authors, without undue reservation.

## Author Contributions

All authors listed have made a substantial, direct and intellectual contribution to the work, and approved it for publication.

## Conflict of Interest

The authors declare that the research was conducted in the absence of any commercial or financial relationships that could be construed as a potential conflict of interest.

## Publisher’s Note

All claims expressed in this article are solely those of the authors and do not necessarily represent those of their affiliated organizations, or those of the publisher, the editors and the reviewers. Any product that may be evaluated in this article, or claim that may be made by its manufacturer, is not guaranteed or endorsed by the publisher.

## Footnotes

1
http://drive5.com/uparse/

2
https://www.arb-silva.de/

## Abbreviations

AA: acetic acid
ADF: acid detergent fiber expressed inclusive of residual ash
aNDF: neutral detergent fiber assayed with a heat-stable amylase and expressed inclusive of residual ash
BA: butyric acid
CK: control
CP: crude protein
DM: dry matter
FM: fresh matter
FW: fresh weight
LA: lactic acid
LAB: lactic acid bacteria
LP: *Lactobacillus plantarum*
NGS: next generation sequencing
OTU: operational taxonomic unit
PA: propionic acid
SEM: standard error of mean
WSC: water soluble carbohydrates.
